# Primary transcriptomes of *Mycobacterium avium *subsp. *paratuberculosis *reveal proprietary pathways in tissue and macrophages

**DOI:** 10.1186/1471-2164-11-561

**Published:** 2010-10-12

**Authors:** Harish K Janagama, Elise A Lamont, Sajan George, John P Bannantine, Wayne W Xu, Zheng J Tu, Scott J Wells, Jeremy Schefers, Srinand Sreevatsan

**Affiliations:** 1 Department of Veterinary Population Medicine, University of Minnesota, 1365 Gortner Avenue, Saint Paul, 55108, USA; 2 Department of Veterinary Biomedical Sciences, University of Minnesota, 1971 Commonwealth Avenue, Saint Paul, 55108, USA; 3 Minnesota Supercomputing Institute, University of Minnesota, 117 Pleasant Street SE, Minneapolis, 5455, USA; 4 National Animal Disease Center, Agricultural Research Service, United States Department of Agriculture, 2300 Dayton Road, Ames50010, USA

## Abstract

**Background:**

*Mycobacterium avium *subsp. *paratuberculosis *(MAP) persistently infects intestines and mesenteric lymph nodes leading to a prolonged subclinical disease. The *MAP *genome sequence was published in 2005, yet its transcriptional organization in natural infection is unknown. While prior research analyzed regulated gene sets utilizing defined, in vitro stress related or advanced surgical methods with various animal species, we investigated the intracellular lifestyle of MAP in the intestines and lymph nodes to understand the MAP pathways that function to govern this persistence.

**Results:**

Our transcriptional analysis shows that 21%, 8% and 3% of the entire MAP genome was represented either inside tissues, macrophages or both, respectively. Transcripts belonging to latency and cell envelope biogenesis were upregulated in the intestinal tissues whereas those belonging to intracellular trafficking and secretion were upregulated inside the macrophages. Transcriptomes of natural infection and in vitro macrophage infection shared genes involved in transcription and inorganic ion transport and metabolism. MAP specific genes within large sequence polymorphisms of ancestral *M. avium *complex were downregulated exclusively in natural infection.

**Conclusions:**

We have unveiled common and unique MAP pathways associated with persistence, cell wall biogenesis and virulence in naturally infected cow intestines, lymph nodes and in vitro infected macrophages. This dichotomy also suggests that in vitro macrophage models may be insufficient in providing accurate information on the events that transpire during natural infection. This is the first report to examine the primary transcriptome of MAP at the local infection site (i.e. intestinal tissue). Regulatory pathways that govern the lifecycle of MAP appear to be specified by tissue and cell type. While tissues show a "shut-down" of major MAP metabolic genes, infected macrophages upregulate several MAP specific genes along with a putative pathogenicity island responsible for iron acquisition. Many of these regulatory pathways rely on the advanced interplay of host and pathogen and in order to decipher their message, an interactome must be established using a systems biology approach. Identified MAP pathways place current research into direct alignment in meeting the future challenge of creating a MAP-host interactome.

## Background

*Mycobacterium avium *subsp. *paratuberculosis *(MAP) causes one of the most well documented chronic diseases of ruminants worldwide (Johne's disease (JD)) and yet the cues leading to its intracellular survival live in obscurity[1]. Major hindrances involved in examining gene regulation during MAP infection are the low amounts of bacterial RNA isolated from an infected host and the lack of an appropriate animal model [2]. In order to overcome the limited quantity of RNA, previous transcriptomic studies interrogating genes used in pathogenic mycobacterial infection were conducted utilizing mimetic conditions of infection in an in vitro environment (i.e. hypoxia, nutrient starvation, acid and nitric oxide (NO) stresses, etc.) [2,3].

While these studies provided insight into a limited number of genes regulated by specific cues, it is not representative of natural infection since mycobacteria will encounter more than one stress at a time. Multiple stressors may change which genes are utilized as well as potential for gene:gene or protein:protein interactions that influence survival and dissemination in the host. Therefore, current investigations into the intracellular fate of MAP and host responses rely on in vitro macrophage models, specifically bovine and murine cells[3-8]. Studies from our laboratory using an in vitro bovine macrophage infection model in conjugation with selective capture of transcribed sequences (SCOTS) revealed upregulation of MAP genes involved in combating oxidative stress, metabolic and nutritional starvation and cell survival at 48 and 120 hrs post infection[9]. These results indicate that common sets of genes are required for MAP to persist within a multifaceted host environment. Furthermore, consistent with another study using SCOTS analysis with *Mycobacterium avium*, MAP expresses several genes involved in fatty acid degradation, which has been suggested as a universal theme used by pathogenic mycobacteria to successfully efface and invade macrophages and other cell types [10-12].

The utility of results from in vitro macrophage infections, as well as small animal models, is controversial as it is currently unknown if these applications faithfully reflect natural infection in MAP's preferred host. A recent study by Meyer-Barber et al. shows discrepant requirements for Toll expression between isolated murine bone marrow derived macrophages from in vitro and in vivo *M. tuberculosis *infection[10]. Since pathogens initiate and inhibit host signaling (i.e. recognition or evasion), there is also a potential for MAP regulatory networks to differ during in vivo infection. Additionally, a number of articles investigating host-MAP interactions use BOMAC (bovine macrophage) cells due to the advantage of having a cell line[13,14]. However, BOMAC cells are inherently dysfunctional; lacking several receptors and possessing an insufficient capability to phagocytose MAP[8,15]. Therefore, macrophage studies to date may 1) underestimate the speed of MAP responses and/or 2) may be serving as an apparition of rather than being an accurate representation of infection. More importantly, in vitro macrophage studies do not address the initial events that set the venue for MAP's transition into the macrophage. Prior to residing inside gut macrophages, MAP must first encounter the intestinal epithelium [1]. The intestinal epithelium represents a formidable fortress that actively secretes IgA and antimicrobial peptides, which is shielded by the glycocalyx and a thick layer of mucus, produced by intestinal goblet cells [16]. Therefore, it is of little surprise that most of the disease signs associated with JD (i.e. transmural inflammation, corrugation, and gross lesions) are inflicted upon the intestinal tissue. Despite MAP's successful siege against the intestinal barrier as evidence of its infiltration into lamina propria macrophages, the exact genes and pathways MAP employs within the intestinal epithelium remains a black hole in our understanding of overall pathogenesis[17]. Furthermore, it has been suggested that MAP processing by the epithelium may aid in efficiency of invasion in macrophages by pre-exposure to a hyperosmolar environment or expression of a MAP oxidoreductase (MAP3464) [17,18]. Thus, it seems short sighted to assume that no disparate mechanisms are used to survive in the intestinal tissue and macrophage given two different cell types with varying function. Furthermore, data compiled by the Immune Epitope Database and Analysis (IEDB) suggest that specific mycobacterial epitopes are present only within a given host. Studies using small animal models, such as the mouse, may not capture a comprehensive MAP epitope profile as well as transcriptome representative of the cow. The elucidation of host-specific epitopes and MAP genes required for survival during natural infection are expected to aid in the rational design of JD vaccines.

The aim of this study was to characterize the functional MAP transcriptional profiles in the ileum and mesenteric lymph node (MLN) of naturally infected cattle as well as an in vitro bovine monocyte derived macrophages (MDMs) infection model. We have employed advanced molecular techniques, computational and bioinformatic analyses to identify and characterize MAP gene expression during the natural infection process.

## Results

### Isolation and identification of MAP

**Figure 1:**
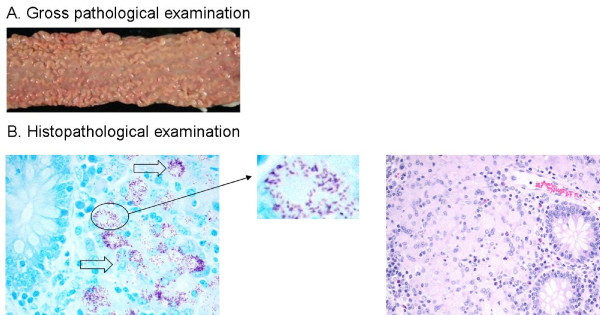
**MAP infection in subclinically infected animals**. (A) Section of bovine ileum infected with MAP: Longitudinal section of ileum showing inflammation and corrugated appearance of inner mucosal layer from a dairy cow with subclinical Johne's disease.(B) Histopathology of bovine ileum with MAP: Acid fast stain (400×) (left) and hematoxylin and eosin stain (100×) (right) of an ileal section of subclinical JD cow in Fig. 1A showing MAP organisms.

Postmortem examination of two subclinical JD cattle revealed gross lesions and corrugation throughout the intestine indicative of chronic inflammation, especially within the ileum (Fig. 1A is a representative example). Histopathological sections of the ileum identified MAP by modified Ziehl-Neelson staining for acid-fast organisms (Fig. 1B), which was later confirmed by standard culture and PCR methods (Table 1). MAP was successfully isolated from intestinal lesion, mesenteric lymph nodes, liver and spleen of both subclinically infected animals. All isolates were genotyped by SSR analysis as > 13G and 5GGT repeats, which was identical to MAP K-10 culture (15G and 5GGT) used for macrophage infection.

**Table 1:** Fecal culture results of MAP isolated from intestinal tissues

Animal ID	Organ	Colony Count	Test Result
386	Ileum	> 100	positive
386	Mesenteric lymp node	> 100	positive
39	Ileum	1-10	positive
39	Mesenteric lymp node	> 100	positive

MAP organisms were grown in Herrold's egg yolk medium for 12 weeks at 37°C. The colonies were counted.

### Gene expression of MAP during natural infection

Analysis of MAP from infected tissues showed differential expression of 2167 genes compared to broth cultures. After multiple test corrections, 1795 genes were significantly different at *q *≤ 0.05 by unpaired t-test. Amongst these, 1684 genes were altered at ≥ 1.5 fold change and 1054 genes at fold change ≥ 2.0 compared with corresponding MAP isolates in broth culture. Table 2 shows a list of operons and Additional file 1 Tables S1, S2 and S3 show complete lists of genes differentially regulated during natural infection.

**Table 2:** List of operons expressed in tissues

Operon	Function
MAP0150c-MAP0152c	Acetyl-coA dehydrogenase
MAP0232c-MAP0237c	Cell wall biosynthesis
MAP0564-MAP0569	MCE family
MAP1778c-MAP1780c	Lipid metabolism
MAP0107-MAP0116	MCE family
MAP2171c-MAP2177c	Mycobactin biosynthesis
MAP3464-MAP3465	ABC transporters
MAP2310c-MAP2314c	Fatty acid degradation
MAP1712-MAP1716	Fatty acid biosynthesis
MAP1522-MAP1523	Fatty acid biosynthesis
MAP2569c-MAP2571c	Glycosyl transferase

**Figure 2:**
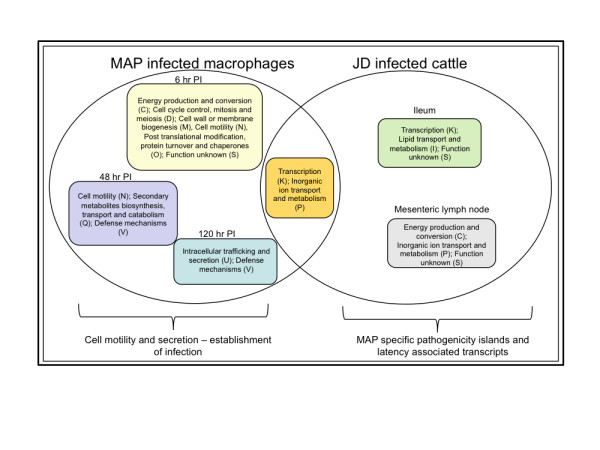
**Classification of differentially expressed MAP genes into Clusters of orthologous genes (COG) groups**. Differentially expressed genes in the tissues or infected macrophages were grouped based on clusters of orthologous genes (COG) classification. Significantly enriched COGs under each condition are represented in the Venn diagram. Shown in the parenthesis is the code for each COG category.

Shared and variable genes between the ileum and MLN are represented in Additional files 1, Tables S2 and S3. Genes were classified into various functional groups based on clusters of orthologous genes (COG) classification and the percent gene expression of each group was calculated. Functional groups enriched in both ileum and MLN belonged to virulence (i.e. MAP1575c, MAP3162c), unknown function or poorly described cellular pathways (i.e. MAP3812c, MAP4269c). Genes belonging to transcription (i.e.: MAP1736, MAP2418) and lipid metabolism and transport (i.e.: MAP0556c, MAP1451) were specifically enriched in the ileum, while energy production and conversion (i.e.: MAP1171, MAP2620c) and inorganic ion transport and metabolism (i.e.: MAP0982c, MAP3141c) were enriched in MLN (Fig. 2).

### Gene expression of MAP in an in vitro macrophage infection assay

A total of 562 MAP genes were differentially expressed during macrophage infection compared with broth culture. Amongst them, 556 genes had a ≥1.5 fold change and 462 genes had ≥ 2.0 fold change (p ≤ 0.05). At 6 hr post infection (PI), upregulated genes of significant interest included serine/threonine protein kinase, *pkn*B (MAP0016c), ATPase, AAA family protein (MAP0167) and PPE family protein (MAP1675). At 48 hour PI MAP upregulated PE family proteins (MAP0140; MAP0339, MAP1507), transcriptional regulators (MAP0475; MAP2428c) and *fad*D27 protein (MAP3156). Finally, at 120 hr PI MAP displayed higher induction of genes concerning major membrane protein, *mmp*L4 (MAP0076, MAP1240c), MCE-family proteins (MAP0566, MAP0759), PE-family proteins (MAP0140; MAP4076), oxidoreductase (MAP0444; MAP3507), lipase, *lip*E (MAP0248) and ABC transporters (MAP0563). A total of 55 genes were shared across different time points in the macrophage infection assay using MAP K-10 strain. Fig. 2 shows the distribution of the differentially expressed genes across three time points and Additional file 1, Tables S4, S5 and S6 shows the detailed list of genes.

### Comparisons of gene expression profiles of naturally infected tissues and in vitro macrophage infection

While a total of 126 genes were commonly expressed between infected tissues and macrophages, 928 and 336 genes were specifically represented in tissues or macrophages, respectively (Fig. 2, Additional File 1, Tables S1 and S7). Functional categories belonging to transcription (MAP1631c, MAP1634, MAP3967) and inorganic ion transport and metabolism (MAP1110, MAP3773c, MAP4171) were represented both in tissues and macrophages (Additional Fig. 2). Macrophage specific gene expression represented functional categories belonging to cell cycle control (MAP2990c), cell wall biogenesis (MAP0670c), cell motility (MAP1506) and secretion (MAP1515). Tissue specific gene expression included genes categorized into virulence mechanisms and those that were not represented in any of the COG groups. Furthermore, MAP regulates expression of persistence related genes such as MAP0033c (WhiB family protein), MAP0038 (probable biofilm regulator), and MAP0075 (mycobacterium specific membrane protein) during natural infection.

### Expression of MAP lineage specific genes during natural infection

Approximately 96 genes distributed in six loci (LSP 4, 11, 12, 14 and 15) were recently described as MAP lineage specific genomic insertions; majority of these genes were consistently upregulated (fold change > 2.0, p < 0.05) in the in vitro infected macrophages whereas downregulated in the tissues of both the animals (Additional File 1, Tables S8 and S9) [19]. Loci of interest include LSP 4 and 11, which carry putative prophages, transposons and unique sequences with no hits in NCBI. MAP0858, located within LSP 4, has conserved domains resembling those of a virulence factor (proteophosphoglycan) belonging to *Leishmania*. LSP11 contains MAP2149c, which has conserved domains to that of SARP (**S**treptomyces **A**ntibiotic **R**egulatory **P**rotein) family transcriptional factor. Located within LSP 12 includes a mammalian cell entry (*mce*) operon (MAP2190 - MAP2194) which was downregulated in the tissues whereas MAP2189 (*mce*) and MAP2180c (a beta lactamase like protein) were upregulated in the macrophages. Downregulated genes located within LSP 14 belong to ABC transporter operon (MAP3731c - MAP3736c), siderophore biosynthesis operon (MAP3741 - MAP3746) and oxidoreductase (MAP3756c). However, an oxidoreductase (MAP3744), and ABC type transporter (MAP3739c) and a polyketides synthase (MAP3763c) belonging to LSP14 were all upregulated in macrophage infections. An ABC transporter operon (MAP3774c - MAP3776c), which is located on LSP 15, was downregulated in infected tissues. Interestingly, MAP3773c, a probable Ferric Uptake Regulator protein on LSP 15, was downregulated in the tissues and upregulated in experimentally infected macrophages. Lastly, located within the LSP specific for cattle MAP strains is an enzyme involved in xenobiotic biodegradation and metabolism (MAP1728c *yfnB*) was downregulated in the tissues whereas upregulated in the in vitro infected macrophages.

### Real-time validation of microarray data

**Figure 3:**
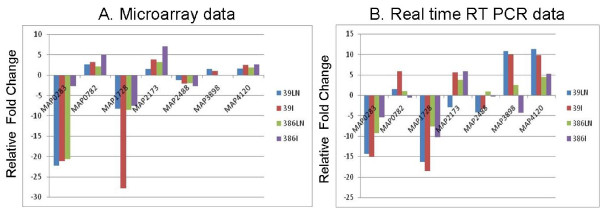
**Comparisons of fold changes of selected genes by microarray and real time RT PCR**. (A) Selected MAP genes that were differentially regulated (up or down) after subtraction with broth culture (data in linear scale). (B) These genes were validated for their expression pattern by real-time PCR to demonstrate similar trends in gene expression (data in logarithmic scale).

We selected seven genes for real-time PCR to validate microarray results. These genes were chosen based on their roles in diverse pathways. Selected genes included membrane protein (MAP0283c), inorganic ion transport (MAP0782, MAP2488), iron acquisition (MAP2173c), energy production and metabolism (MAP3898, MAP4120) and finally an LSP specific for cattle strains of MAP (MAP1728c). RNA extracts used for microarray analysis (ileum, MLN and macrophages) were also analyzed for their level of expression by real-time PCR assay with primers designed using universal probe library (Roche, Indianapolis, IN). The expression of these genes in the tissues of JD cows shows the same trend in microarray and the real-time analyses. MAP K-10 broth culture was used as a control to determine fold change. Fig. 3 demonstrates the fold change ratios of selected MAP genes in the microarrays as compared to their gene expression in real-time after normalization with a housekeeping gene *secA*.

## Discussion

The hallmark of MAP infection is the subclinical manifestation of a persistent intestinal infection. Yet, surprisingly, there remains a paucity of studies investigating the intracellular lifestyle of MAP in the intestinal epithelium in comparison to research involving macrophage and/or lymphocyte models [4,9]. We sought to fill this critical knowledge gap by reporting the first transcriptome analysis of MAP in infected tissues and macrophages. Both the ileum and mesenteric lymph node have been suggested to act as potential MAP reservoirs within the host; therefore, it is critical to understand the MAP pathways that function to govern this persistence [1,20,21]. The current trend in MAP research is to isolate and analyze regulated gene sets given defined, in vitro stress related cues or during a particular infection stage using surgical methods and various animal species [3,22,23]. However, we have taken a more directed approach to uncover common and unique pathways utilized by MAP in intestinal tissues using the natural host under natural infection. Elucidation of the transcriptome active in local infection sites is expected to not only augment our knowledge of MAP pathogenesis, which will lend itself to the establishment of a host-pathogen interactome, as well as rational design of vaccines and/or antimycobacterial therapeutic modalities.

### MAP residing within the ileum is primed for persistence in subclinical infection

Pathogenic mycobacteria have the uncanny ability to persist within the host for an indefinite period of time that can last several years[24,25]. Although the genes and signals that induce persistence remain unclear, mycobacteria entering this phase are characterized by a state of chronic or prolonged non-replication [24]. One cue that primes the cell to enter into the non-replication stage is the stringent response, which is characterized by the *relA *controlled production of hyperphosphorylated guanosine ((p)ppGpp) activated upon nutrient deprivation, hypoxia and oxidative stress [26,27]. Together *relA *and (p)ppGpp are able to combat hostile environments by negatively regulating bacterial "life" signals such as DNA and protein machinery. Interestingly, we have identified a unifying theme from naturally infected host tissue as the downregulation of several energy, carbohydrate, amino acid and lipid metabolism as well as transcriptional and DNA replication related genes. Similar attributes of the stringent response were found to be selectively upregulated within the ileum. For example, the RelA/SpoT domain-containing protein has a three-fold upregulation in the ileum. Recently Geiger and colleagues have shown that the RelA/SpoT domain-containing protein, RSH synthetase/hydrolase enzyme, in *Staphylococcus aureus *is responsible for maintained production of (p)ppGpp and concomitant repression of genes regulating translational machinery [28]. Furthermore, a single metabolism gene regulating menaquinone biosynthesis and consequent production of vitamin K (MAP4052) was uniquely upregulated in the ileum[29]. In addition to initiating the synthesis of mycobactins, menaquinone biosynthesis genes have been shown to be a critical factor in maintaining non-replicating mycobacterial cell viability[30].

Stringent response priming of MAP cells is most likely due to host inflicted stresses, particularly nitric oxide resultant in DNA damage[26]. Previous studies examining MAP "scrapings" from the intestinal wall of JD clinical cattle show significant upregulation of *katG*, a bacterial catalase gene used to combat oxidative stress[31]. Furthermore, granulomatous lesions within the ileum or lymph node isolated from cattle naturally infected with either MAP or *M. bovis*, respectively, have enhanced immune-staining for natural resistance-associated macrophage protein 1 (NRAMP1) and inducible nitric oxide synthase (iNOS), which together synthesize nitric oxide [32,33]. Although we did not identify enrichment of *katG *in the ileum, we show upregulation of MAP2836, a LexA repressor, which is stimulated upon DNA damage and stress and results in the arrest of cell division and induction of DNA repair[34]. Similarly, increase in a LysR transcriptional regulator (MAP2442) within the ileum is indicative of an oxidative stress response[35]. These data suggest that during the early stages of infection, MAP is primed for persistence by the stringent response in order to avoid oxidative stress and DNA damage. This appears to be a "watershed moment" in the intracellular lifecycle of MAP as persistence during subclinical infection will ensure its survival and future dissemination into other organs.

### MAP evades immune detection in the MLN

Similar to MAP pathways found in the ileum, the majority of MAP genes involved in energy, carbohydrate, inorganic ion, DNA repair, transcription and translation pathways are downregulated. However, there is a lack of stringent response as well as persistence-associated expression. The MLN contains populations of circulating effector cells, such as T and B cells; therefore, MAP may downregulate the aforementioned pathways to avoid detection by the host immune system[16]. Furthermore, common to both ileum and MLN, MAP upregulates several genes associated with cell envelope and outer member biogenesis (MAP1905c, MAP3019c and MAP3979). It is well established that mycobacterial cell wall components have immunomodulatory functions that enable pathogenic mycobacteria to escape immune surveillance by suppression of pro-inflammatory cytokines, phagosome-lysosome fusion and MHC class II presentation[5,36-38]. Thus, MAP may surround itself with complex cell wall associated glycolipids to prevent recognition and continue t unabated by the host immune system.

### Expression of lineage specific large sequence polymorphisms (LSPs) during natural and in vitro macrophage infection

Comparative genomics of the *M. avium *complex (MAC) revealed that MAP evolved as a pathogen by acquiring large segments of DNA (i.e. pathogenicity islands) via horizontal gene transfer [19,39-41]. Our study is the first to directly show that some of these putative pathogenicity islands are associated with virulence. Contrary to expression found within the tissues, genes belonging to the LSPs were upregulated in macrophage infection. Qt-RT-PCR analysis also demonstrated that MAP1728c (*yfnB*), a gene involved in xenobiotic biodegradation and metabolism located within the LSP (deletion 2) specific for cattle MAP strains was downregulated in the tissues [19]. This is consistent with the regulation of other MAP genes, which suggests that MAP transcriptional machinery remains silent in the tissues. Several iron related genes were downregulated in tissues including LSP15, a MAP unique pathogenicity island that encodes a ferric uptake regulator (MAP3773c), as well as the ABC transporter (MAP3731c - MAP3736c), and a possible siderophore biosynthesis operon (MAP3741-MAP3746) that contains a FUR binding box within the intergenic region [42]. This is of significant interest as part of this region (MAP3731c-MAP3736c) has previously been shown to be immunogenic and preliminary studies indicate its use as a potential vaccine candidate[43]. Furthermore, our genome analysis revealed that a type VII secretory system (esx3) was located immediately downstream of LSP15. Esx3 has recently been shown to be essential for mycobactin synthesis and we have identified its repression by the MAP iron dependent regulator (IdeR) in the presence of iron [44,45]. Taken together, transcriptional analysis suggests that LSP14, 15 and esx-3 form a major pathogenicity island that may play a potential role in maintaining iron homeostasis and hence survival within the macrophage.

## Conclusions

MAP is an extremely resilient pathogen that employs a number of regulatory pathways to ensure its survival. Regulatory pathways that govern the lifecycle of MAP appear to be specified by tissue and cell type. While tissues show a "shut-down" of major MAP metabolic genes, infected macrophages upregulate several MAP specific genes along with a putative pathogenicity island responsible for iron acquisition. Despite differences in gene programs found within tissues and cell types, the overriding rule of MAP is to progress by deception either by entering a persistent state, shielding by complex cell wall components or hiding in the macrophage. Many of these programs rely on the advanced interplay of host and pathogen and in order to decipher their message, an interactome must be established using a systems biology approach [25,46]. Preliminary interactomes for the current study are reminiscent of those being developed for *S. pyogenes *and *H. pylori *and show promising networks that may aid in our understanding of overall pathogenesis as well as potential targets for novel vaccines and therapeutics [47,48]. The findings presented in this study will lend themselves in meeting this future challenge of creating a MAP-host interactome.

## Methods

All cattle work in this study was performed according to institutional guidelines and approved animal care and use protocols at the University of Minnesota.

### Sampling from subclinical JD cows

Two sub-clinically infected but apparently healthy dairy cattle, identified as low shedders by routine serological and fecal culture methods at the University of Minnesota Veterinary Diagnostic Laboratory, were purchased from a farmer and euthanized for this study. The infection status of the animals were established using standard serology for MAP-specific antibody (Idexx Laboratories, Inc., Westbrook, ME) [49] and fecal culture[50]. At necropsy, sections from affected portions of the intestines ileum, ileocecal junctions, and the surrounding enlarged mesenteric lymph nodes (MLN) were harvested, wrapped in aluminum foil and either snap-frozen in liquid nitrogen or fixed in formalin for RNA extraction and histopathological examination, respectively. All samples were stored at -80.0°C until RNA extraction. Furthermore organs were triturated and cultured for of the presence MAP using standard mycobacterial culture techniques. Sections of the MLN and ileum were taken for microscopy using hematoxylin and eosin staining and acid fast staining. A total of seven slides were created and imaged for each stain.

### Genotyping of MAP

MAP colonies were sub-cultured in Middlebrook 7H9 broth (MB7H9) (DIFCO, Lawrence, KS) supplemented with oleic acid-albumin-dextrose-catalase (OADC) enrichment medium (Fischer Scientific, Inc., Pittsburgh, PA) and mycobactin J (2 mg/L) (Allied Monitor, Inc., Fayette, Missouri) at 37°C with subtle shaking. MAP isolates were determined free of contaminant bacteria by absence of growth on Brain-Heart Infusion (BHI) agar at 37°C. Following genomic DNA extraction using a standardized protocol (Qiagen, Valencia, CA), isolates were confirmed for MAP specific IS*900 *insertion sequence by PCR and agarose gel electrophoresis. MAP isolates from infected tissues as well as MAP cattle strain K-10 (MAP K-10) were genotyped based on short sequence repeats (SSR) from two polymorphic (G and GGT) loci as described [48,49].

### Macrophage infection assay

Monocyte derived macrophages (MDMs) were prepared using a previously described method [4,51]. Briefly, blood was collected from the jugular vein of a JD-free healthy cow and mixed with an equal portion of acid-citrate dextrose to prevent coagulation. Blood was transferred in 40 mL aliquots into DNase/RNAse free conical tubes and centrifuged at 2,200 rpm for 20 min. at room temperature. Buffy coats were separated, washed in 1X D-PBS and layered on a 58% percoll gradient (Sigma-Aldrich, St. Louis, MO). Cells were collected from percoll, washed 1X PBS and expanded in RPMI containing 20 percent autologous serum at 37°C in 5 percent CO_2_. MDMs were allowed to incubate for 4 days prior to seeding. MDMs were subsequently seeded at ~2.0 × 10^7 ^cells/flask in 25 cm2 flasks and allowed to adhere for 2 hr. A seed stock of MAP K-10 was sub-cultured and grown to mid-logarithmic growth phase (OD_600 _= 1.0) in MB7H9 broth (supplemented with OADC enrichment medium and 2 μg/ml of mycobactin J) at 37°C on a shaker set at 120 rpm. MAP K-10 was used at a 20:1 multiplicity of infection (MOI) in all infections. Infection was conducted in RPMI containing 2% autologous serum. Following infection after 2 hr,, MDMs were washed twice with fresh, pre-warmed serum-free RPMI 1640 (Gibco(r) Invitrogen, Inc., Carlsbad, CA) to remove non-adherent bacteria and the cultures were subsequently grown in RPMI 1640 with 2% autologous serum for 6, 48 and 120 hrs in duplicate at each time point.

### Nucleic acid extraction

Prior to RNA extraction, all surfaces and equipment were treated with RNAse Away (Molecular Bioproducts, Inc., San Diego, Inc.). For total RNA extraction, ~30 mg of mesenteric lymph nodes and ileum were ground separately in liquid nitrogen using a mortar and pestle and dissolved in 600 μL of RLT buffer (Qiagen Inc., Valencia, CA). Total RNA from infected MDMs (6, 48 and 120 hrs p.i,) and MAP K-10 broth cultures were extracted by TRIzol reagent (Invitrogen Inc., Carlsbad, CA) per manufacturer's instructions. Samples were homogenized in a mini bead-beater (Biospec) with 0.3 ml of 0.1 mm sterile RNase-free zirconium beads for 4 min. followed by RNA extraction using RNeasy (Mini) kit (Qiagen Inc., Valencia, CA). All samples were treated with RNase-free DNase I (Ambion, Inc., Austin, TX) to eliminate genomic DNA contamination. The purity and yield of total RNA samples was examined using Nanodrop spectrophotometer (Thermo Scientific Inc., Wilmington, DE) and Agilent 2100E Bioanalyzer (Agilent Technologies, Inc., Santa Clara, CA). Purity of RNA samples were validated by the absence of MAP locus 251 amplification via PCR. All samples were stored at -80°C until later analysis.

### Enrichment and confirmation of MAP transcripts

Total RNA obtained from naturally infected tissues and experimentally infected MDMs were processed to remove host RNA as well as ribosomal RNA. Similarly, the total RNA from broth cultures of tissue isolates and MAP K-10 were enriched for bacterial messenger RNA by removing ribosomal RNA. All samples were subjected to RNA amplification and analyzed on a regular denaturing agarose gel and Agilent 2100 bioanalyzer (Agilent Technologies, Santa Clara, CA). Furthermore, the presence of MAP specific genes was confirmed using RT-PCR, sequencing and BLAST analyses (data not shown) prior to use in microarrays.

### Sample processing and microarray hybridizations

All microarray experiments were conducted using the minimal information about a microarray experiment (MIAME) guidelines. Polyadenylated host mRNA and bacterial rRNA were eliminated by processing the samples with MICROBEnrich and MICROB*Express *Bacterial mRNA Purification Kits (Ambion Inc., Austin, TX), respectively. RNA samples were amplified using MessageAmp(tm) II-Bacteria Kit for prokaryotic RNA amplification system (Ambion Inc., Austin, TX) and labeled with SuperScript(tm) Plus Direct cDNA Labeling System (Invitrogen Inc., Carlsbad, CA). MAP transcripts from infected tissues (two sections each for ileum and mesenteric lymph node) and macrophage infection assay (performed in duplicates) were combined individually with sheared genomic DNA of MAP K-10 labeled with BioPrime(r) Plus Array CGH Genomic Labeling System (Invitrogen Inc., Carlsbad, CA) and hybridized onto 70-mer oligonucleotide microarrays (obtained from Dr. Michael Paustian, NADC, Iowa). Every predicted open reading frame in the MAP strain K-10 genome is represented on this array. One 70-mer was designed for each gene with a total length of less than 4000 bp, while longer genes were split in half and one 70-mer oligo was designed for each half. Additional details of this microarray design can be found elsewhere[52]. RNA from MAP K-10 broth culture and tissue isolates was also processed in the same manner. After overnight hybridization, microarray slides were washed and scanned using the HP Scanarray 5000 (PerkinElmer Inc., Waltham, MA). Images were collected and stored for expression analyses. Microarray experiments were repeated twice for each sample.

### Microarray data analysis

Numeric data was extracted from the two-channel hybridization images using the microarray image analysis software, BlueFuse (BlueGnome Ltd, Cambridge). Following normalization by global lowess, the gene expression data was analyzed by GeneSpring GX 10.0 (Agilent Technologies Inc., Foster city, CA). Two group T test was performed to identify the differentially expressed MAP genes (DEGs) and multiple test correction was applied to the T test. The DEGs in natural infected tissues (ileum and mesenteric lymph nodes) and in vitro infected macrophages were identified after normalizing the data with MAP in broth culture. The lists of genes obtained from the above were analyzed using Basic Local Alignment Search Tool (BLAST) algorithm in National Center for Biotechnology Information (NCBI) database against the MAP K-10 genome and the 11 mycobacterial genomes listed in the NCBI databank. Gene IDs were categorized into various functional groups based on Clusters of Orthologous Groups (COGs). Differentially regulated genes were also uploaded in Pathway Studio 6.0 (Ariadne genomics Inc., Rockville, MD) with the *M. tuberculosis *H37Rv database to explore the cellular context of differentially expressed genes by computational methods of protein network identification.

### Quantitative Real-time PCR validation

Selected genes from microarray data were validated using two-step SYBR-green based quantitative real-time PCR (Roche, Indianapolis, IN) analysis in Roche LightCycler 480 II (Roche Inc., Indianapolis, IN). Primers were designed using web-based tools, Primer3 http://frodo.wi.mit.edu/primer3/ or Universal Probe Library (Roche Inc., Indianapolis, IN) and verified by BLAST searches to confirm their specific binding to target sequences (Table 3). The following cycle program was used: denaturation at 95°C for 15 min. and PCR at 95°C for 10 s, 65°C for 15 s, 72°C for 22 s for 55 cycles. RNA (ileum, MLN and macrophage) used in microarray analysis was also used in real-time PCR. MAP K-10 broth culture served as a control for all RNA samples. Test and control samples were normalized using the house keeping gene, *secA*, and relative expression was calculated by 2^-ΔΔ*C*T ^method [53]. Results are reported as fold change. Each sample was conducted in triplicate.

**Table 3:** Primer sequences used in Q-RT PCR

Gene and direction	Sequence
MAP0233c, F	ggggtagaaggacaggaagc
MAP0233c, R	agttctacgccagcatcgac
MAP0283, F	caatcttccgggtctaccac
MAP0283, R	gagccggtactgatggtga
MAP0782, F	ttcgtgtgcctgtgcaac
MAP0782, R	gcgacttcgttggtggtc
MAP1728, F	cagccacaaatacgacatcc
MAP1728, R	gtgacgaaggctgtttgga
MAP2173c, F	gcagggtgcggtagtgac
MAP2173c, R	ccgagtatctggtcgaggtg
MAP2488, F	gccggttgctcaactacct
MAP2488, R	tcaggcagaacgtcaggaa
MAP3698, F	ccgtcgatgtaccaccagt
MAP3698, R	catcggctccttggtgat
MAP4120c, F	ggaaaccaagggatgtcgt
MAP4120c, R	acgagacgctgcaagagc
*secA*, F	ggcctgctccttgaggtt
*secA*, R	gcgcaaggtgatctacgc

## Authors' contributions

SS conceived the idea. HKJ, EAL and SS analyzed the data and wrote the manuscript. SG performed experiments and analyzed the microarray data. WWX and JZT helped in microarray data analysis and bioinformatic analysis. JBP and SJW contributed to new reagents. JS performed necropsy, histopathology and microbiological culture. All authors read and approved the manuscript.

## Acknowledgements

We would like to thank the Microbial and Plant Genomics Institute, biomedical genomics Center and Computational genetics Laboratory at the University of Minnesota for providing resources and services to perform the studies.

## Supplementary Material

Additional file 1 **MAP identified genes in ileum, mesenteric lymph node and in vitro infected bovine macrophages**. Fold changes, putative functions and regulation of MAP genes uniquely identified and shared in the ileum, mesenteric lymph node and in vitro infected bovine macrophages. Tables: S1-S9. S1: Ileum specific MAP genes. S2: Mesenteric lymph node specific MAP genes. S3: Ileum and Mesenteric lymph node shared MAP genes. S4: Macrophage specific MAP genes (6 and 48 hrs PI). S5: Macrophage specific MAP genes (6 and 120 hrs PI). S6: Macrophage specific MAP genes (48 and 120 hrs PI). S7: Common MAP genes between natural and in vitro infection. S8: Expression of MAP lineage specific LSPs in the tissues of naturally infected cattle. S9: Expression of MAP lineage specific LSPs in the in vitro infected macrophages. Click here for file

Additional file 2 **Pathway analysis of COGs enriched in tissues and macrophages**. COGs enriched in tissues or macrophages were used to identify interactions with other groups and their diverse roles in various cellular processes using Pathway Studio 6.0 (Ariadne genomics Inc., Rockville, MD). Pictorial representation of the interactions of (A) Lipid metabolism genes centered on *kasA *(MAP 1998), a cell wall biogenesis gene upregulated in the tissues and (B) Intracellular trafficking and secretion genes centered on PE_PGRS4, a PPE family gene upregulated in macrophages. kasA interacts with other proteins such as pknL (MAP1914) and plays a role in lipid metabolism and cell survival. PE_PGRS4 interacts with other proteins such as prrC, rpiA and plays a role in colonization and virulence. Green ovals indicate metabolites, red ovals indicate genes and gold rectangles indicate processes. Click here for file
