# Supplementary figures and images for: Primary transcriptomes of Mycobacterium avium subsp. paratuberculosis reveal proprietary pathways in tissue and macrophages
Source: BMC Genomics. 2010 Oct 12;11:561. doi: 10.1186/1471-2164-11-561 (PMC3091710; doi:10.1186/1471-2164-11-561)

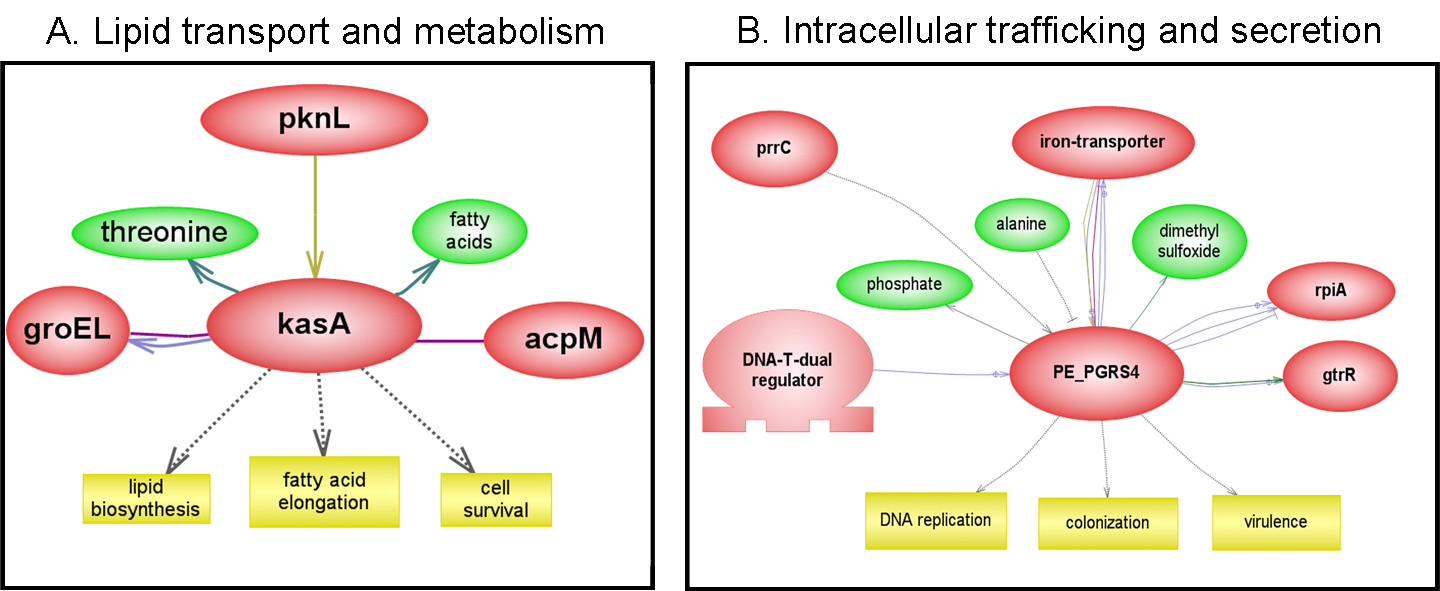

Supplement: Additional file 2 — Pathway analysis of COGs enriched in tissues and macrophages. COGs enriched in tissues or macrophages were used to identify interactions with other groups and their diverse roles in various cellular processes using Pathway Studio 6.0 (Ariadne genomics Inc., Rockville, MD). Pictorial representation of the interactions of (A) Lipid metabolism genes centered on kasA (MAP 1998), a cell wall biogenesis gene upregulated in the tissues and (B) Intracellular trafficking and secretion genes centered on PE_PGRS4, a PPE family gene upregulated in macrophages. kasA interacts with other proteins such as pknL (MAP1914) and plays a role in lipid metabolism and cell survival. PE_PGRS4 interacts with other proteins such as prrC, rpiA and plays a role in colonization and virulence. Green ovals indicate metabolites, red ovals indicate genes and gold rectangles indicate processes. [file 1471-2164-11-561-S2.TIFF]
